# Clinical outcomes of sharp esophageal foreign bodies in elderly patients: a retrospective study from Wuhan, China

**DOI:** 10.3389/fmed.2025.1653609

**Published:** 2025-10-28

**Authors:** Jiahang Xu, Baojun Chen, Yong Liu

**Affiliations:** Department of Thoracic Surgery, The Central Hospital of Wuhan, Tongji Medical College, Huazhong University of Science and Technology, Wuhan, Hubei, China

**Keywords:** esophagus, foreign body, elderly, endoscopy, surgery, outcomes

## Abstract

**Objective:**

This study aimed to explore the clinical characteristics, treatment determinants, and outcomes of elderly patients with sharp esophageal foreign bodies (EFBs) in Wuhan, China.

**Method:**

We conducted a retrospective analysis of 58 elderly patients (≥65 years) with sharp EFBs treated at Wuhan Central Hospital from January 2017 to June 2023. Patients were allocated to either an endoscopic (*n* = 43) or surgical (*n* = 15) treatment group based on clinical severity. We analyzed demographics, EFB type, injury severity, pre-hospital delay, laboratory findings, length of hospital stay, and functional outcomes using the Barthel Index. Statistical comparisons, including effect sizes and 95% confidence intervals, were performed, and a multivariable logistic regression analysis was conducted to identify independent predictors for surgical intervention.

**Results:**

No significant differences were observed in baseline demographics (*p* > 0.05). Fish bones (60.3%) were the most common EFB. Factors significantly associated with requiring surgical treatment included longer pre-hospital delay (median 3.0 vs. 0.5 days, *p* < 0.001), presence of fever (60.0% vs. 4.7%, *p* < 0.001), leukocytosis (46.7% vs. 16.3%, *p* = 0.031), dysphagia (20.0% vs. 2.3%, *p* = 0.041), and pre-existing esophageal diseases (53.3% vs. 14.0%, *p* = 0.004). All patients in the surgical group had esophageal perforation or a peri-esophageal abscess, compared to only 25.6% in the endoscopic group (*p* < 0.001). Multivariable analysis identified pre-hospital delay (OR 2.55, 95% CI [1.29–5.04]) and presence of fever at admission (OR 15.8, 95% CI [3.01–82.9]) as independent predictors for surgery. Endoscopic treatment was associated with a significantly shorter hospital stay (mean 4.3 vs. 13.7 days, *p* < 0.001) and superior functional recovery at discharge (*p* = 0.008) and 1 month post-procedure (*p* < 0.001).

**Conclusion:**

Delayed medical consultation, severe complications like perforation and abscess, and underlying esophageal comorbidities are key factors necessitating surgical intervention for sharp EFBs in the elderly. Fever at admission and pre-hospital delay are strong independent predictors of the need for surgery. Prompt diagnosis and endoscopic management, when feasible, are associated with shorter hospitalizations and better functional outcomes.

## Introduction

Esophageal foreign body (EFB) is a common emergency in otolaryngology and thoracic surgery. Delayed diagnosis or improper treatment can cause serious complications, including death (1, 2). The causes of EFB ingestion are multifactorial and closely related to the patient’s age, eating habits, mental status, and underlying diseases (3, 4). Additionally, pre-existing esophageal lesions, such as stenosis, cancer, or motility disorders, increase the risk of impaction. EFBs are particularly prevalent in the elderly and children (5, 6). Elderly individuals often experience cognitive decline, tooth loss, weakened swallowing functions, and decreased mucosal sensation, making them susceptible to accidental ingestion of foreign objects like dislodged dentures or poorly chewed food (7). While children typically ingest blunt objects, sharp EFBs in adults are more likely to damage the esophagus, causing perforation and other severe complications, thus requiring more urgent treatment (8).

The complications of EFBs arise from direct injury to the esophagus and adjacent structures in the neck, chest, and mediastinum (9). The spectrum of complications is wide, with the most feared being an aortoesophageal fistula, which can cause fatal hemorrhage (10). Therefore, a thorough understanding of the clinical characteristics of EFBs and prompt, accurate diagnosis are crucial for effective treatment and improved prognosis. This article presents a retrospective study of the clinical features, treatment modalities, and outcomes of sharp EFBs in elderly patients in the Wuhan area, aiming to provide a theoretical basis for optimizing diagnosis and treatment selection in this vulnerable population.

## Materials and methods

### Patient population

**Figure 1:**
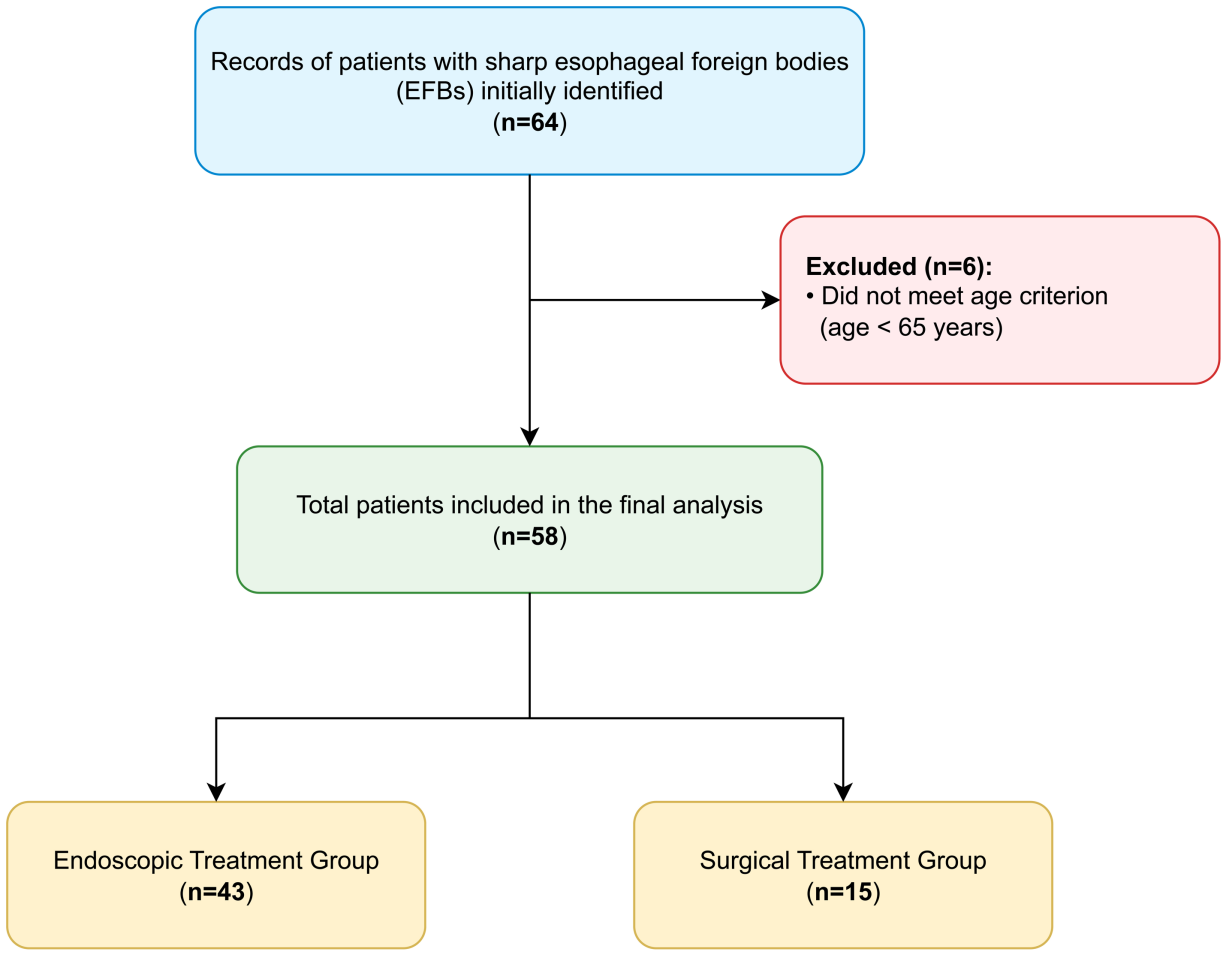
Flowchart of patient selection for the study.

We retrospectively reviewed the clinical data of patients admitted with sharp EFBs to Wuhan Central Hospital between January 2017 and June 2023. A patient flowchart is provided in Figure 1. Inclusion criteria were: (1) Patients aged ≥65 years old. (2) Diagnosis of a sharp EFB (defined as a hard object with sharp edges) confirmed by digestive endoscopy or computed tomography (CT) scan after admission. (3) The foreign body remained impacted in the esophagus during treatment. (4) The patient or their family provided consent for treatment. Exclusion criteria were: (1) No EFB identified on imaging or endoscopy. (2) Patient withdrew from or refused treatment. All patients or their families in this study signed informed consent forms. This study was reviewed and approved by the Ethics Committee of Wuhan Central Hospital (No. WHZXKYL2023-185).

### Study groups and data collection

After screening, a total of 58 patients were included in the final analysis. Patients were divided into an endoscopic treatment group (*n* = 43) and a surgical treatment group (*n* = 15) based on their presenting clinical severity and the presence of complications.

### Diagnostics

Upon presentation, a detailed history of foreign body ingestion was recorded. All patients underwent diagnostic imaging to confirm the diagnosis. In our cohort, 5 patients (8.6%) underwent a barium meal examination, all 58 patients (100%) had a CT scan, and 53 patients (91.4%) underwent digestive endoscopy for diagnosis or treatment. Body temperature and white blood cell count were monitored to assess for infection. Based on imaging, endoscopy, and surgical findings, the degree of esophageal injury was classified as mucosal injury, ulcer, perforation, or peri-esophageal abscess.

### Treatments

The decision to proceed with endoscopic or surgical treatment was made by a multidisciplinary team including the attending thoracic surgeon and endoscopist based on a comprehensive clinical evaluation. Generally, endoscopic removal was the first-line approach for all patients. Surgical intervention was indicated for patients who met one or more of the following criteria, indicating severe clinical conditions: (1) Radiological evidence of esophageal perforation with extraluminal contamination (e.g., mediastinitis, para-esophageal abscess, or significant pneumomediastinum); (2) Clinical signs of sepsis or hemodynamic instability; (3) Failure of endoscopic removal, defined as the inability to safely dislodge and extract the foreign body without causing further esophageal injury; or (4) High suspicion of major vascular injury based on preoperative CT imaging. All endoscopic procedures were performed by senior endoscopists with at least 10 years of experience, using standard flexible gastroscopes (Olympus GIF-Q260J). The choice of endoscopic removal tool (e.g., foreign-body forceps, snare, or basket) was at the discretion of the endoscopist based on the EFB’s shape, size, and location. To ensure safe extraction and protect the esophageal mucosa and pharynx from injury during retrieval, a protective overtube was used at the discretion of the endoscopist, particularly for larger or irregularly shaped sharp objects. Endoscopic failure, which necessitated conversion to surgery, was documented in cases where the foreign body could not be visualized, grasped, or safely removed without significant risk of mucosal tearing or perforation, as judged by the attending endoscopist. For patients undergoing successful endoscopic treatment, post-procedural management depended on the extent of esophageal injury. Patients with minor mucosal damage were managed with a liquid diet, while those with suspected or contained perforation without significant infection received nasogastric tube feeding. Surgical treatment involved foreign body removal, esophageal repair, debridement of abscesses, and placement of drainage tubes, with additional interventions as needed for complications.

### Analysis indicators

The analysis indicators included: (1) Basic patient information: age, gender, comorbidities. (2) Admission-related indicators: EFB type, degree of esophageal injury, fever (axillary temperature >37.3 °C), white blood cell count (normal range: 3.5–9.5 × 10^9^/L), time from onset to admission, hospital stay, and pain score (Visual Analogue Scale). Pre-existing esophageal-related diseases included conditions such as esophageal stenosis, a history of esophageal cancer (post-treatment, no active disease), or severe reflux esophagitis. Abnormal swallowing function was primarily identified by the clinical symptom of dysphagia, as reported in the patient’s history. (3) Quality of life comparison: We compared the functional status (Barthel Index total score) between the two groups at admission, at discharge, and 1 month post-procedure. The Barthel Index was used to assess the impact of the event and treatment on patients’ functional independence in activities of daily living (ADL), a critical outcome in geriatric populations. While not a direct measure of disease-specific quality of life, it provides valuable insight into the broader functional recovery (11).

### Statistical analysis

Data analysis was conducted using SPSS 23.0 software. Normally distributed data are expressed as mean ± standard deviation (Mean ± SD) and compared using an independent sample *t*-test. Non-normally distributed data are represented by the median (interquartile range, IQR) and compared using the Mann–Whitney U test. Count data are expressed as *n* (%) and compared using the chi-square test or Fisher’s exact test. Effect sizes were calculated: odds ratios (OR) with 95% confidence intervals (CI) for categorical data, Cohen’s d for *t*-tests, and Rosenthal’s r for non-parametric comparisons. To identify independent predictors of requiring surgical treatment, a binary logistic regression analysis was performed. Variables that were statistically significant in the univariate analysis (*p* < 0.05) were included in the multivariable model. A *p*-value <0.05 was considered statistically significant.

## Results

### Types of foreign bodies

**Figure 2:**
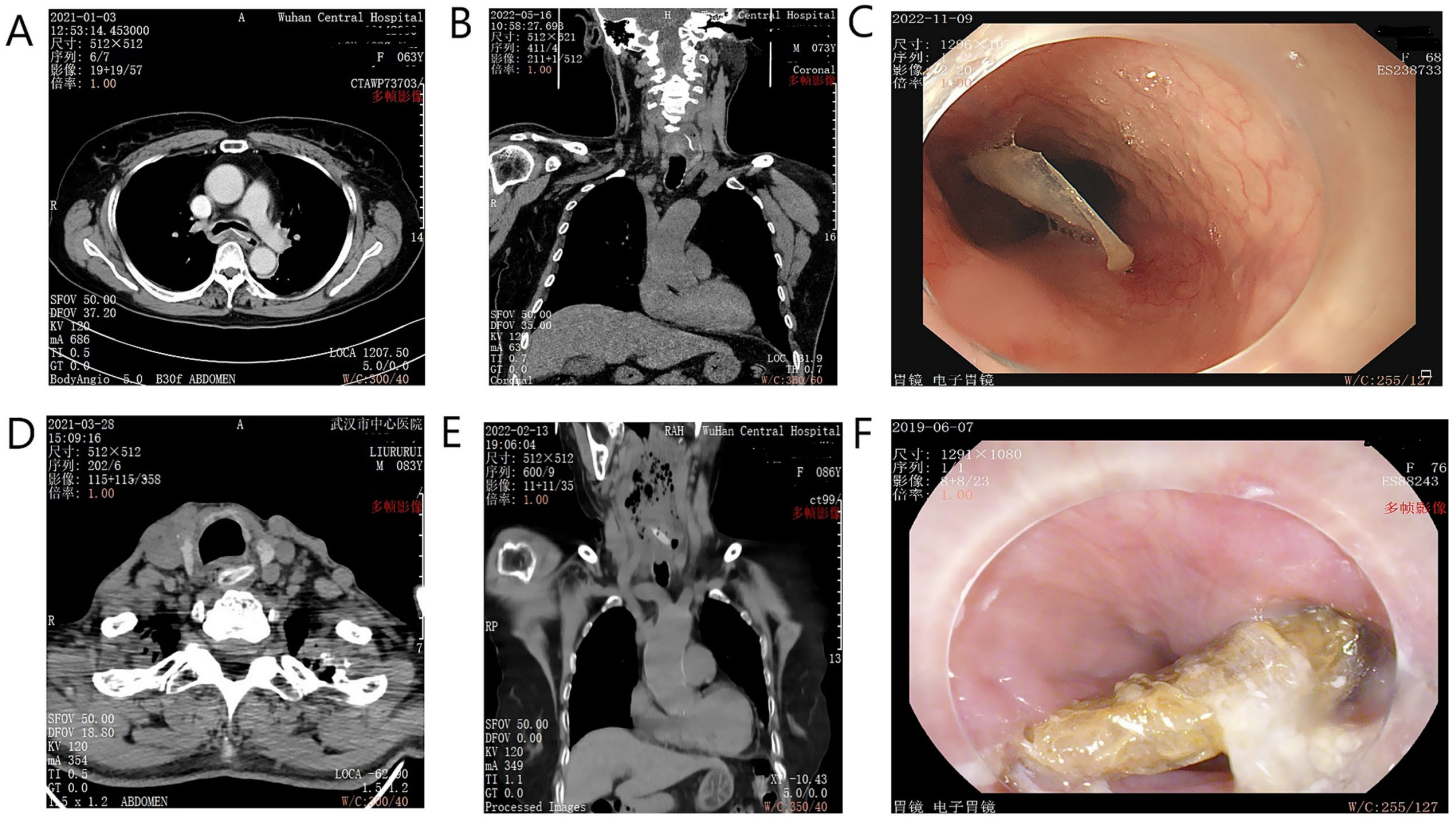
Examples of sharp foreign bodies identified by CT and endoscopy. **(A)** Axial CT scan showing a fish bone penetrating the esophageal wall. **(B)** Coronal CT view of a similar injury. **(C)** Endoscopic view of an impacted fish bone. **(D)** Axial CT showing a jujube pit in the cervical esophagus. **(E)** Coronal CT view of an impacted jujube pit causing peri-esophageal inflammation. **(F)** Endoscopic view of a large, impacted jujube pit.

The types of sharp objects included jujube pits, fish bones, bullfrog bones, poultry bones, and pig bones. Fish bones (*n* = 35, 60.3%) and jujube pits (*n* = 15, 25.9%) were the most common. The incidence rates of bullfrog bones, poultry bones, and pig bones were 2 (3.4%), 4 (6.9%), and 2 (3.4%), respectively (examples are shown in Figure 2).

### Baseline characteristics and clinical presentation

The baseline characteristics of the 58 patients are compared in Table 1. There were no significant demographic differences between groups (*p* > 0.05). However, patients in the surgical group had a significantly longer median pre-hospital delay compared to the endoscopic group [3.00 (IQR 1.00, 4.00) days vs. 0.50 (IQR 0.19, 1.00) days; *p* < 0.001]. A significantly higher proportion of patients in the surgical group presented with fever (60.0% vs. 4.7%; *p* < 0.001) and an abnormal white blood cell count (46.7% vs. 16.3%; *p* = 0.031) at admission.

**Table 1:** Comparison of baseline demographic and clinical characteristics between the endoscopic and surgical treatment groups.

Index	Endoscopic treatment group (*n* = 43)	Surgical treatment group (*n* = 15)	Statistic (Z/χ^2^)	*p*-value	Effect size (OR/r) [95% CI]
Gender (Female, %)	25 (58.1)	11 (73.3)	0.981	0.431	1.94 [0.48, 7.78]
Age (years, median [IQR])	70.0 [67.0, 80.0]	68.0 [66.0, 71.0]	−0.512	0.609	–
Time from onset to admission (days, median [IQR])	0.50 [0.19, 1.00]	3.00 [1.00, 4.00]	−3.651	**<0.001**	−0.48
Fever upon admission (Yes, %)	2 (4.7)	9 (60.0)	18.411	**<0.001**	29.25 [5.41, 158.1]
WBC count upon admission (abnormal, %)	7 (16.3)	7 (46.7)	4.640	**0.031**	4.36 [1.20, 15.82]
Residence (city, %)	14 (32.6)	7 (46.7)	0.864	0.353	1.81 [0.55, 5.96]
Education level (junior high or less, %)	28 (65.1)	14 (93.3)	4.321	0.109	–

Data are presented as *n* (%), median (IQR), or mean ± SD. Statistical comparisons were made using the Mann–Whitney U test, chi-square test, or Fisher’s exact test. OR, Odds Ratio; CI, Confidence Interval; r, Rosenthal’s r effect size for Mann–Whitney U test. *p*-values in bold are statistically significant.

### Differences in EFB location, injury severity, and treatment

Sharp object impaction occurred most frequently in the upper esophagus in both groups, with no significant difference in location (*p* > 0.05) (Table 2). The median pain score was significantly higher in the surgical group [3.00 (IQR 3.00, 3.00)] compared to the endoscopic group [2.00 (IQR 1.00, 3.00); *p* = 0.003]. The effect size (Rosenthal’s r = −0.39) indicates a medium-sized effect, where the negative correlation signifies that the surgical group (coded as the higher value group) was associated with higher pain scores. There was a significant difference in esophageal injury severity: all 15 patients (100%) in the surgical group had esophageal perforation or a peri-esophageal abscess, compared to only 11 of 43 patients (25.6%) in the endoscopic group (*p* < 0.001) (Table 2). Among the 15 surgical cases, 5 underwent right thoracotomy, 7 required a cervical incision after failed endoscopic attempts, and 3 had other surgical approaches.

**Table 2:** Differences in EFB location and injury severity between the two patient groups.

Index	Endoscopic treatment group (*n* = 43)	Surgical treatment group (*n* = 15)	Statistic (Z/χ^2^)	*p*-value	Effect size (r)
EFB position			0.311	0.856	–
Upper esophagus	28 (65.1%)	10 (66.7%)			
Middle esophagus	14 (32.6%)	5 (33.3%)			
Lower esophagus	1 (2.3%)	0 (0%)			
Pain score (VAS, median [IQR])	2.00 [1.00, 3.00]	3.00 [3.00, 3.00]	−2.940	**0.003**	−0.39
Damage degree (Perforation/Abscess, %)	11 (25.6%)	15 (100%)	20.342	**<0.001**	-

Data are presented as *n* (%) or median (IQR). *p*-values in bold are statistically significant.

### Correlation of dental status and comorbidities with treatment selection

There were no significant differences in dental status between the groups (*p* > 0.05) (Table 3). However, comorbidities were strongly associated with treatment selection. Patients requiring surgery were significantly more likely to have pre-existing esophageal-related diseases (53.3% vs. 14.0%; *p* = 0.004) or present with dysphagia (20.0% vs. 2.3%; *p* = 0.041) compared to those managed endoscopically (Table 4).

**Table 3:** Comparison of dental status between the two patient groups.

Index	Endoscopic treatment group (*n* = 43)	Surgical treatment group (*n* = 15)	Statistic (Z/χ^2^)	*p*-value
Number of teeth (median [IQR])	22.0 [12.0, 26.0]	24.0 [18.0, 28.0]	−0.731	0.465
Tooth loss (Yes, %)	37 (86.0%)	11 (73.3%)	1.237	0.268
Fixed partial denture (Yes, %)	17 (39.5%)	6 (40.0%)	0.003	0.955
Removable dentures (Yes, %)	13 (30.2%)	2 (13.3%)	1.758	0.317

Data are presented as *n* (%) or median (IQR).

**Table 4:** Comparison of comorbidities and dysphagia between the two patient groups.

Index	Endoscopic treatment group (*n* = 43)	Surgical treatment group (*n* = 15)	Statistic (χ^2^)	*p*-value	OR [95% CI]
Esophageal-related comorbidities (Yes, %)	6 (14.0%)	8 (53.3%)	8.211	**0.004**	6.67 [1.81, 24.51]
Dysphagia (Yes, %)	1 (2.3%)	3 (20.0%)	4.175	**0.041**	10.64 [1.02, 110.6]

Data are presented as *n* (%). *p*-values in bold are statistically significant.

### Multivariable analysis of predictors for surgical treatment

To identify independent predictors for requiring surgical intervention, a binary logistic regression analysis was performed. The variables included were pre-hospital delay, fever at admission, abnormal WBC count, dysphagia, and pre-existing esophageal disease. The analysis, summarized in Table 5, revealed that a longer pre-hospital delay (OR 2.55, 95% CI [1.29–5.04]; *p* = 0.007) and the presence of fever at admission (OR 15.8, 95% CI [3.01–82.9]; *p* = 0.001) were significant independent predictors for the necessity of surgical treatment. Other factors did not retain statistical significance in the multivariable model.

**Table 5:** Multivariable logistic regression analysis of predictors for surgical treatment.

Variable	Odds ratio (OR)	95% confidence interval (CI)	*p*-value
Pre-hospital delay (per day increase)	2.55	1.29–5.04	**0.007**
Fever at admission (Yes vs. No)	15.8	3.01–82.9	**0.001**
Abnormal WBC count (Yes vs. No)	2.10	0.52–8.45	0.298
Pre-existing esophageal disease (Yes vs. No)	3.45	0.88–13.5	0.075
Dysphagia (Yes vs. No)	4.02	0.35–46.1	0.261

WBC, White Blood Cell. *p*-values in bold are statistically significant.

### Post-treatment outcomes and follow-up

All patients were discharged without major complications. As shown in Table 6, endoscopic treatment was associated with a significantly shorter hospital stay (mean 4.30 vs. 13.73 days; mean difference −9.43, 95% CI for difference [−12.50, −6.36]; *p* < 0.001, Cohen’s d = −2.18). While functional status at admission was similar between groups (*p* = 0.429), the endoscopic group had significantly higher Barthel Index scores at discharge (mean 89.53 vs. 76.00; mean difference 13.53, 95% CI [3.78, 23.28]; *p* = 0.008) and at 1 month post-procedure (mean 100.00 vs. 97.00; mean difference 3.00, 95% CI [1.88, 4.12]; *p* < 0.001). At the 6-month follow-up, four patients in the surgical group reported minor residual symptoms (e.g., occasional dysphagia or retrosternal discomfort), compared to two in the endoscopic group. All symptomatic patients underwent a follow-up gastroscopy, which revealed mild esophageal mucosal inflammation without evidence of stenosis or residual foreign bodies; they were managed conservatively with proton pump inhibitors.

**Table 6:** Comparison of post-treatment outcomes between the two patient groups.

Index	Endoscopic treatment group (*n* = 43)	Surgical treatment group (*n* = 15)	Statistic (*t*)	*p*-value	Effect size (Cohen’s d) [95% CI for difference]
Duration of hospitalization (days, mean ± SD)	4.30 ± 2.05	13.73 ± 6.20	−8.912	**<0.001**	−2.18 [−12.5, −6.3]
Barthel Index Score (mean ± SD)					
At admission	70.12 ± 24.51	75.00 ± 19.63	−0.796	0.429	–
At discharge	89.53 ± 14.89*	76.00 ± 20.02	2.845	**0.008**	0.79 [3.8, 23.3]
1 month after discharge	100.00 ± 0.00*	97.00 ± 4.55*	4.701	**<0.001**	1.31 [1.9, 4.1]

Data are presented as mean ± SD. *p < 0.05 compared with the score at admission within the same group. *p*-values in bold are statistically significant.

## Discussion

Management of sharp EFBs in the elderly presents a significant clinical challenge due to age-related comorbidities and the high risk of severe complications. This study provides valuable insights by identifying key predictors for surgical intervention in this vulnerable population. Our principal finding is that pre-hospital delay and the presence of fever on admission are strong, independent predictors of the need for surgery, a conclusion strengthened by our multivariable analysis. These findings underscore the critical importance of early presentation and diagnosis in improving outcomes.

This study has several limitations that must be acknowledged. As a single-center, retrospective analysis, it is susceptible to selection bias. The small sample size, especially in the surgical group (*n* = 15), limits the statistical power for detecting smaller effect sizes and compromises the robustness of the multivariable analysis, underscoring the need for validation in larger, multicenter cohorts. The use of the Barthel Index, while useful for assessing general functional recovery, is not a specific measure of post-esophageal intervention quality of life. Despite these limitations, our study provides valuable clinical insights into managing a high-risk condition in a vulnerable population.

EFBs are common in the elderly for several reasons, including dulled mucosal sensation, poor dentition leading to inadequate chewing, and underlying esophageal or neurological conditions that impair swallowing (11–13). Timely removal is the cornerstone of EFB management. Endoscopic removal is the preferred first-line treatment due to its minimally invasive nature and high success rate, reported to be between 92.5 and 98.9% (1, 14, 15). However, for patients with severe complications like large perforation, mediastinitis, or systemic sepsis, surgery becomes necessary. Choosing the appropriate treatment is especially critical in elderly patients to minimize morbidity.

Our data strongly suggest that the primary drivers for surgical intervention are the development of severe complications like perforation and infection. This was confirmed by our finding that all patients in the surgical group had perforation or abscess and is further supported by our multivariable analysis, which identified pre-hospital delay and fever as strong, independent predictors of needing surgery. Delayed presentation is a critical factor; prolonged impaction (>24 h) is a known risk factor for esophageal perforation, with complication rates increasing significantly over time (16, 17). Our findings align with this, showing that delayed medical attention leads to a higher likelihood of complications necessitating surgery.

Pre-existing esophageal diseases and dysphagia were also associated with surgical treatment in our univariate analysis. Elderly patients with underlying swallowing difficulties may not recognize the symptoms of an EFB, leading to delayed diagnosis until severe symptoms like odynophagia develop, by which time a perforation may have already occurred. In contrast, dental status had little impact on treatment selection in our cohort, likely because the main EFBs were small items like fish bones and jujube pits rather than large dentures (18, 19).

Endoscopic treatment is the preferred modality for uncomplicated EFBs due to its safety and efficacy (20, 21). Recent guidelines continue to emphasize early endoscopic intervention to prevent the progression to more severe complications that would mandate surgical intervention (22). Surgery is reserved for complex cases with complications like abscesses or major vessel injury (23). While life-saving, surgical treatment is associated with longer hospitalization, higher costs, and more significant postoperative pain, as demonstrated in our study.

The predominance of fish bones and jujube pits in our study is likely linked to local dietary habits in Wuhan and may not be generalizable to other regions. Provided there are no contraindications, early endoscopy should be performed to remove such objects. Even in cases of small perforations without abscesses, endoscopic management followed by conservative care can be sufficient.

## Conclusion

Delayed medical consultation, severe complications such as perforation and abscess formation, and pre-existing esophageal conditions are key determinants that necessitate surgical intervention for sharp EFBs in the elderly population. Fever at admission and longer pre-hospital delay are strong independent predictors of the need for surgery. Prompt diagnosis and endoscopic management, when clinically appropriate, are associated with significantly shorter hospital stays, reduced morbidity, and better functional outcomes. Therefore, we recommend prompt CT evaluation and endoscopic intervention for elderly patients with suspected sharp EFBs to prevent complications. Future large-scale, prospective studies are warranted to confirm these predictors and explore the utility of specific quality-of-life instruments in this population.

## Data Availability

The original contributions presented in the study are included in the article/supplementary material, further inquiries can be directed to the corresponding author.
